# Optimal Timing for Resuming Antithrombotic Agents and Risk Factors for Delayed Bleeding after Endoscopic Resection of Colorectal Tumors

**DOI:** 10.1155/2014/825179

**Published:** 2014-12-07

**Authors:** Kazuko Beppu, Taro Osada, Naoto Sakamoto, Tomoyoshi Shibuya, Kenshi Matsumoto, Akihito Nagahara, Takeshi Terai, Tatsuo Ogihara, Sumio Watanabe

**Affiliations:** Department of Gastroenterology, Juntendo University School of Medicine, 2-1-1 Hongo, Bunkyo-ku, Tokyo 113-8421, Japan

## Abstract

*Aim*. To examine optimal timing for resuming antithrombotic agents and risk factors for delayed bleeding after endoscopic resection of colorectal tumors. *Method*. Of 1,970 polyps larger than 10 mm removed by polypectomy, endoscopic mucosal resection, or endoscopic submucosal dissection, delayed bleeding, which was designated as bleeding that occurred 6 or more hours after endoscopic treatment, occurred in 52 cases (2.6%); 156 nonbleeding cases matched for age and gender were controls in this single-institution retrospective case-control study. We investigated (1) patient-factors: resuming antithrombotic agents within 5 days following endoscopic resection, hypertension, and diabetes mellitus; and (2) tumor-factors: morphology, size, location, and resection technique by conditional logistic regression. *Results*. By multivariate analysis resumption of anticoagulants within 5 days was a significant risk factor for delayed bleeding (OR 10.2; 95% CI = 2.7–38.3; *P* = 0.0006). But resuming a thienopyridine within 5 days was not (OR 0.9; 95% CI = 0.1–2.6; *P* = 0.40). Other patient- and tumor-factors were not significant. *Conclusion*. Resuming anticoagulants within 5 days after endoscopic treatment was associated with delayed bleeding whereas resuming thienopyridines was not.

## 1. Introduction

While endoscopic resection is the standard method of treatment for colorectal tumors and highly effective in reducing the incidence of colon cancer and its subsequent mortality [1, 2], it is associated with a substantial risk of complications. The most common major complication is hemorrhage, which is reported to occur in 0.3% to 6.1% [3, 4] of patients. Mortality is uncommon, but the possible need for recolonoscopy, blood transfusion, and sometimes embolization makes delayed postpolypectomy hemorrhage a potentially serious complication.

Several factors such as large polyp size and sessile form have been reported to increase the risk of bleeding after endoscopic treatment, but controversies still exist [5–11]. In our prior study [12], we found that tumors larger than 10 mm were a risk factor for delayed bleeding after endoscopic resection. At present, there are limited data in the literature about risk factors focusing on these higher risk polyps. In addition, whether resuming anticoagulants and antiplatelet agents after endoscopic resection increases the risk of delayed bleeding has become a question of vital concern over the past decade, particularly with the increasingly widespread use of these drugs. However, little to no data on this subject has been publicly available. Furthermore, the optimal timing for restarting antithrombotic agents after endoscopic treatment is unknown.

We performed a case-control study to evaluate the risk factors including patient and tumor characteristics associated with bleeding after endoscopic treatment.

## 2. Method

### 2.1. Patients

**Figure 1:**
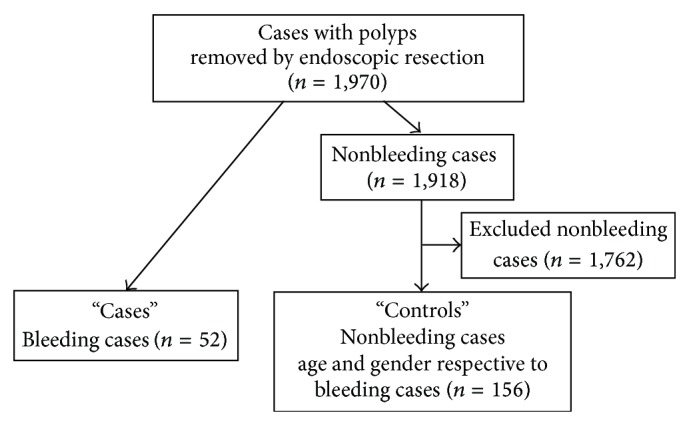
Flow diagram showing the process for analysis in the case-control study.

From January 2006 to October 2012, 1,970 cases with polyps of the colon and rectum, measuring larger than 10 mm, were removed by polypectomy, endoscopic mucosal resection (EMR), or endoscopic submucosal dissection (ESD) at our hospital. All procedures were performed by 5 expert endoscopists. Patients were instructed to contact the endoscopist immediately if they had bloody feces. We identified patients who reported delayed bleeding by examining in patients' records for the 30-day period after the procedure and checking for hospital visits to ensure that we did not overlook any cases of delayed bleeding in this study. “Cases” were defined as those patients who developed delayed bleeding, which was designated as bleeding that occurred 6 or more hours after endoscopic treatment. For such cases, after admission, second-look colonoscopy was performed to identify the origin of bleeding, and endoscopic hemostasis was performed when active bleeding was found. “Controls” were defined as patients who underwent endoscopic treatment with colonoscopy but did not develop delayed bleeding and were matched for age and gender with cases. Controls were selected in a 3 : 1 ratio compared with cases (Figure 1).

### 2.2. Data Collection

We investigated patient-factors (resuming anticoagulants (warfarin, heparin, and dabigatran) and antiplatelet agents (aspirin and thienopyridines) within 5 days following endoscopic resection, hypertension, and diabetes mellitus) and tumor-factors (morphology, size, location, and resection technique) by univariate and multivariate logistic regression analysis. If anticoagulants or antiplatelet agents had been prescribed, they were discontinued at least 5 days before the procedure. To reduce the risk of thromboembolic events, patients on warfarin and who were at high thromboembolic risk were switched to a shorter-acting therapy—unfractionated heparin—(i.e., bridge) in the periendoscopic period according to guidelines [13]. Morphology of polyps was divided into two types: pedunculated and sessile. Polyp morphology was classified using the criteria of the Japanese Research Society Classification (JRSC) [14]: sessile (Is) polyps were defined as raised lesions without evidence of a stalk or pedicle in which the diameter did not exceed twice the height. Pedunculated (Ip) polyps referred to lesions with an identifiable stalk. Polyp size was also divided into two groups: ≥20 mm and <20 mm. Location of polyps was divided into two areas: left side (rectum to descending colon) and right side (transverse colon to cecum). Resection technique was divided into two types: polypectomy and EMR/ESD. Polypectomy techniques involved the use of snares to remove the polyps with frequency electric current. EMR/ESD techniques also involved the use of snares or a knife with high frequency current. Patients undergoing cold biopsy and cold polypectomy were not included in this study. Among the bleeding cases, we also studied the intervals and median days from endoscopic treatment to bleeding.

### 2.3. Statistics

Statistical analysis was performed using SPSS version 19.0 for Windows. The correlation between delayed bleeding and risk variables was assessed by a two-sided Fisher's exact test. In addition, standard logistic regression methodologies were used to calculate the relative risks as odds ratios (ORs) with 95% confidence intervals (CIs). The median intervals between endoscopic treatment and bleeding in cases with and without anticoagulants were compared with the Mann-Whitney *U* test. For all tests, a *P* value of <0.05 was considered statistically significant.

## 3. Results

During the study period, a total of 1,970 cases with colorectal polyps were found and polyps were resected by polypectomy, EMR, or ESD. Of these, 52 cases (2.6%) met the study criteria for delayed bleeding, with 50 requiring endoscopic hemostasis and 2 blood transfusion. During the perioperative period in this study, there were no thrombogenic events. An additional 156 cases were selected, as described above, to serve as controls. Polyps in the case and control group were resected by polypectomy (62 cases), EMR (130 cases), and ESD (16 cases). Baseline characteristics of cases and controls are summarized in Table 1. The cases and controls were both aged 59.5 ± 11.6 (mean ± SD) and 85% were male.

Table 2 shows the measures of association between patient-factors and the risk of delayed bleeding using univariate analysis. Antithrombotic agents that were used were warfarin (13 cases), heparin bridging therapy (7 cases), dabigatran (1 case), aspirin (17 cases), and thienopyridines (10 cases). Anticoagulants (warfarin and/or dabigatran and/or heparin) were resumed within 5 days following endoscopic resection in 23% of bleeding cases, compared with 6% of the controls (OR 4.9; *P* = 0.0003). Aspirin was not found to be a significant risk factor for delayed bleeding (OR 1.7; *P* = 0.31). Resuming thienopyridines within 5 days after endoscopic resection was not found to be a significant risk factor for delayed bleeding (OR 1.8; *P* = 0.37). Also, hypertension or diabetes mellitus was not found to increase the risk for delayed bleeding.

Results of univariate analysis of tumor-factors for delayed bleeding are shown in Table 3. Resection technique (OR 1.1; *P* = 0.86), polyp size (OR 1.3; *P* = 0.47), morphology (OR 1.4; *P* = 0.28), and polyp location (OR 0.9; *P* = 0.80) were not found to increase the risk for delayed bleeding.

Multivariate analysis of factors influencing the bleeding revealed that a significant factor was resuming anticoagulants within 5 days after endoscopic resection (OR 10.2; 95% CI 2.7–38.3; *P* = 0.0006). Resuming aspirin and thienopyridines, the resection technique and tumor size were not found to be risk factors for delayed bleeding (Table 4).

**Figure 2:**
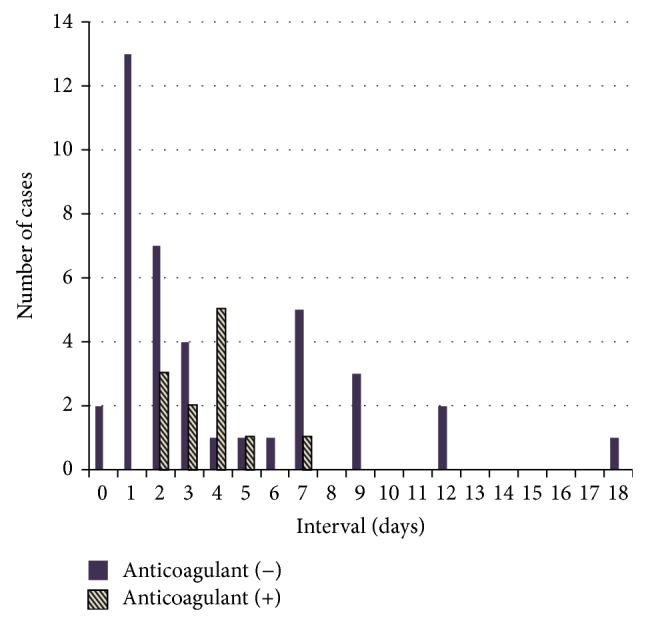
Days from treatment to bleeding in cases who were and were not taking anticoagulants. Delayed bleeding occurred within 7 days in most cases (87%).

Figure 2 shows the days from endoscopic treatment to delayed bleeding in cases who were and were not taking anticoagulants. Of the 52 bleeding cases, 12 were taking anticoagulants, which included 5 using heparin bridging therapy, and 40 were not taking anticoagulants. When examining all cases of delayed bleeding, the bleeding occurred within 7 days in most cases. In exploratory analysis, we compared the median days from endoscopic treatment to bleeding (Table 5). Anticoagulants were resumed within 3 days in 9 of 12 cases who had been taking anticoagulants and from3 to 5 days in the remaining 3 cases. The median days was significantly longer in cases taking anticoagulants than in those who were not (median = 4 days versus 2 days, *P* = 0.04).

## 4. Discussion

The use of anticoagulants and antiplatelet medications for various cardiovascular and hematologic conditions has become increasingly widespread over the past decade [15]. As these medications are thought to potentiate bleeding, endoscopists must weigh the risk of thromboembolic events resulting from the interruption of antithrombotic therapy against the risk of postoperative hemorrhage before performing endoscopic procedures on patients taking these medications. Nevertheless, the exact parameters for performing endoscopic procedures on these patients are vague, and when to resume these medications is a question of recent concern and calls for further investigation. Therefore, we investigated whether resuming these medications after a procedure poses a risk for bleeding.

This study revealed that resuming anticoagulants within 5 days after endoscopic resection is an independent risk factor and is strongly associated with severe delayed bleeding, whereas resuming antiplatelet agents within 5 days has no association. It can be hypothesized that these results are associated with the mechanism of hemostasis: anticoagulants work on the secondary hemostasis process, such as the manufacturing of fibrin, while antiplatelet agents work on the primary hemostasis process, such as the cohesion of platelets. The former process is stronger, so anticoagulants, which prevent the secondary hemostasis process, tend to enable easy bleeding and thus pose a greater risk for delayed bleeding.

Our practice for resumption of anticoagulants following endoscopic treatment is to restart warfarin and/or heparin within 5 days. Anticoagulants were resumed within 3 days in most cases who had been taking anticoagulants. Our data suggests that resuming anticoagulants within 3 days of endoscopic treatment may result in an increase in the risk of delayed bleeding. We propose taking precautionary hemostatic measures after endoscopic treatment, as this may be effective in preventing delayed bleeding in these cases.

In our study, antiplatelet agents were resumed within 5 days after treatment, and there was no incremental increase in the risk of bleeding with antiplatelet agents, including aspirin. It may be appropriate, however, to consider that all subjects in this study were of Japanese ethnicity. In a study conducted by Lee et al. [16], the opinion on restarting aspirin after polypectomy differed between Eastern and Western endoscopists, with the former restarting aspirin 1 to 3 days after polypectomy and the latter restarting aspirin on the same day. Both restarted antiplatelet agents such as ticlopidine and clopidogrel within 7 days. It is possible that this difference lies in patient demographics: thromboembolism has a higher incidence among Caucasians than Asians; hence, Eastern endoscopists may invest greater concern in the risk of bleeding than that of thromboembolic events, and vice versa. Nevertheless, regardless of ethnicity, the timing for resuming anticoagulants and antiplatelet agents must be determined based on each patient's risks related to the underlying disease.

Based on our results, in cases involving thienopyridines, we can restart this medicine within 5 days after treatment with greater confidence regardless of thromboembolic risk. In contrast, in cases with low thromboembolic risk, as determined by United States guidelines [13], such as those that involve deep vein thrombosis, we should avoid resuming anticoagulants within 5 days after treatment. On the other hand, in cases with high thromboembolic risk, such as those that involve atrial fibrillation associated with valvular heart disease, we should resume anticoagulants as soon as bleeding stability allows, keeping in mind the increased risk of delayed bleeding. It is necessary for medical institutions to minimize the bleeding risk associated with endoscopic procedures while simultaneously minimizing the thromboembolic risk of withdrawing medications. We should provide clear guidelines for the appropriate management of anticoagulants and antiplatelet medications for the duration of endoscopic treatment.

Although our data showed no correlation between polyp location and risk of bleeding, Buddingh et al. [17] suggested that polyp location in the right hemi-colon may be an independent risk factor for delayed bleeding. They hypothesized that fresh ileal fluids containing digestive enzymes and bile acids may dissolve the clot that covers and protects the postpolypectomy ulcer, thus leading to delayed bleeding. This discrepancy between their conclusion and the present study's may be due to the resection technique. Whereas Buddingh et al. described using polypectomy and EMR, the present study used polypectomy, EMR, and ESD. Additionally, the number of cases in our study was relatively limited. An additional number of cases comparable to that of previous studies must be gathered for further analysis.

When bleeding occurs, it is usually recognized within 7 days [18, 19]. Our study suggests that all patients, including but not limited to those taking anticoagulants, should be cautioned regarding the possibility of delayed bleeding as long as 7 days after the procedure. Based on our results, it is generally recommended that endoscopists advise patients to take extra precautions, such as changes in diet, avoiding rigorous exercise, and limiting or abstaining from alcohol consumption for 1 week after endoscopic resection, as reported previously [9, 20]. Particularly in cases taking anticoagulants, we should be aware of an increased risk of delayed bleeding approximately 4 days after endoscopic treatment. A possible reason for this is the delayed effect of warfarin, which can take up to 3 to 5 days to reach full effect. Thus, as time passes, the bleeding risk increases.

Our study had a number of limitations. First, this study was conducted retrospectively in the single institution. A further study to prospectively assess the long-term outcome will be necessary to overcome this limitation. Second, most data in our study were collected before the setting of new Japanese guidelines for gastroenterological endoscopy in patients undergoing antithrombotic treatment [21]. In these guidelines, aspirin is no longer discontinued in patients at high risk of a thromboembolism before these procedures. A new study collecting data from cases in whom these guidelines were implemented is warranted. Third, this study focused on thienopyridines and the number of cases using a thienopyridine was small; thus, results of the analysis may have been inadequate. A further study on a larger scale will be needed. Finally, this study excluded cases utilizing cold biopsy and cold polypectomy, which are becoming more commonly used in Japan and should be included in future research.

In summary, resuming anticoagulants within 5 days after endoscopic treatment was associated with delayed bleeding whereas resuming thienopyridines within 5 days after endoscopic treatment was not. In addition, patients should be monitored for a minimum of 7 days following endoscopic treatment.

## Figures and Tables

**Table 1:** Characteristics of study subjects.

	Cases (*n* = 52)	Controls (*n* = 156)
Age, years (mean ± SD)	59.5 ± 11.6	59.5 ± 11.6
Male, sex, *n* (%)	44 (85)	132 (85)

**Table 2:** Univariate analysis of patient-factors for delayed bleeding.

	Cases (%)	Controls (%)	Odds ratio	*P* value
Resuming anticoagulants	12 (23)	9 (6)	4.9	0.0003^*^
Resuming aspirin	6 (12)	11 (7)	1.7	0.31
Resuming thienopyridines	3 (15)	7 (4)	1.8	0.37
Hypertension	16 (31)	43 (28)	1.2	0.66
Diabetes mellitus	10 (19)	22 (14)	1.5	0.37

**Table 3:** Univariate analysis of tumor-factors for delayed bleeding.

	Cases (%)	Controls (%)	Odds ratio	*P* value
Morphology (pedunculated)	36 (69)	95 (61)	1.4	0.28
Polyp size (≥20 mm)	16 (31)	40 (26)	1.3	0.47
Polyp location (left side)	32 (62)	99 (63)	0.9	0.80
Resection technique (EMR, ESD)	37 (71)	109 (70)	1.1	0.86

Left side: rectum-descending colon.

Right side: transverse colon-cecum.

**Table 4:** Multivariate analysis of risk factors associated with delayed bleeding.

	Odds ratio	95% CI	*P* value
Resuming anticoagulants	10.2	2.7–38.3	0.0006^*^
Resuming aspirin	0.6	0.1–2.4	0.43
Resuming thienopyridine	0.9	0.1–2.6	0.40
Resection technique	1.2	0.5–2.6	0.71
Tumor size	1.5	0.7–3.1	0.29

**Table 5:** Median days from endoscopic treatment to bleeding.

Anticoagulants	+	−	*P* value

Median number of days (range)	4 (2–7)	2 (0–18)	0.04^*^
